# First-Year Students’ Initial Motivational Beliefs at University: Predicted by Motivational Beliefs Derived from Within and Out-of-School Experience and Malleable Regardless of the Extent of Students’ Out-of-School Experience

**DOI:** 10.3389/fpsyg.2017.01258

**Published:** 2017-07-25

**Authors:** Julia Gorges

**Affiliations:** Department of Psychology, Bielefeld University Bielefeld, Germany

**Keywords:** academic self-concept, college students, expectancy-value theory, intrinsic task value, non-traditional students, vocational education and training

## Abstract

The present study tested how academic self-concept of ability (ASC) and intrinsic task value (ITV) transpose onto novel university programs that depart from traditional subject areas within the framework of expectancy-value theory. The study focused on two potential sources of information used to anticipate one’s ASC and ITV regarding new learning content (here: business administration). First, students’ experiences from secondary school, especially their ASCs and ITVs established in a school subject they consider similar to business administration—mathematics—should predict their business administration-specific ASC and ITV. Second, students may have gained relevant experience in out-of-school settings such as internships with business companies or commercial vocational training prior to entering higher education. ASC and ITV developed from out-of-school experiences was hypothesized to predict students’ business administration-specific ASC and ITV as well. However, the likely mismatch between anticipated and actual experience with new contents should lead to revisions of ASC and ITV after entering university reflected in a presumably lower stability compared to secondary school settings. In addition, the extent of students’ out-of-school experience might act as a moderator. Data were collected from 341 first-year students in higher education in Germany before they began their study program and again 3–4 months later. Confirmatory factor analyses support the discriminant validity of the measures used in the study. Results from structural equation modeling show that students’ ASC/ITV derived from relevant out-of-school experience make an important contribution to their initial business administration-specific ASC and ITV beyond their mathematics-specific ASC/ITV. Furthermore, both business administration-specific ASC and ITV showed significantly lower stability coefficients over the initial study phase than research from secondary school indicating revisions to them via experience. Multiple-group structural equation modeling showed no moderating effect of the extent of students’ out-of-school experience. The discussion focuses on interpretations of the expectancy-value theory that explicitly include motivational beliefs derived from out-of-school settings as antecedents of expectancy and value. With respect to practical implications, results are discussed in the light of student counseling and support to help students develop an adequate picture of a study program’s learning contents and overcome initial motivational setbacks.

## Introduction

Widening of participation and increasing the number of graduates in higher education is a prominent agenda item of educational policy makers in most industrial countries (OECD, 2016). From a micro-level perspective on participation in education, a person’s motivation is crucial for choosing from the potential range of learning opportunities. Drawing on the prominent expectancy-value model of achievement-related choices (Wigfield and Eccles, 2000), findings from longitudinal research across high school years indicate that expectancy of success and subjective task value are key determinants of educational task choice. These expectancies and values are highly subject-specific and become relatively stable over time (Denissen et al., 2007; Marcoulides et al., 2008; Archambault et al., 2010; Musu-Gillette et al., 2015). Furthermore, regarding the same subjects and contents, expectancies and values developed throughout the primary and secondary school years predict educational task choice in the transition from high school to higher education (Shernoff and Hoogstra, 2001; Malgwi et al., 2005; Watt, 2006; Nagy et al., 2008; Guo et al., 2015; Musu-Gillette et al., 2015). Therefore, the present study considers individuals’ expectancies and task values—also referred to as motivational beliefs in this paper—underlying choice in higher educational contexts.

After compulsory schooling the range of subjects available for individuals to pursue broadens dramatically. Hence, the narrow set of compulsory subjects from secondary school and before do not map directly onto the spectrum of further and higher educational tasks (German Rectors’ Conference [HRK], 2014; Schröder, 2015). Individuals often face novel academic tasks after leaving compulsory school because study programs in higher education may not share a common label or set of learning contents with the former (Moogan et al., 1999). Because individuals may lack established expectancies and task values regarding these novel learning contents, individuals’ expectations and task values might require significant updating or face reductions leading to dropout risk. In such cases, Gorges and Kandler (2012) suggest that individuals generalize expectancies and task values for novel learning contents based on those established in ostensibly similar learning contents from their previous schooling leading to anticipation of certain motivational beliefs. Because such anticipated expectancies and task values at most qualify as an initial best guess about individuals’ self-concepts and valuation of tasks, individuals probably revise these after gaining personal experience with the novel learning contents. In fact, apparently falsely anticipated expectancies and values are among the most frequently reported reasons for higher education dropout (Stinebrickner and Stinebrickner, 2009; Heublein and Wolter, 2011).

In addition to individuals’ experience from within-school settings, their experience in—and thus motivational beliefs developed from—out-of-school activities relevant to the new educational tasks such as vocational training or internships may contribute to anticipating motivational beliefs regarding novel learning contents. Furthermore, self-concept and task value derived from relevant (i.e., with respect to the novel learning contents) out-of-school experience might boost the accuracy of individuals’ anticipated motivational beliefs. However, given that the extent of individuals’ relevant out-of-school experience likely varies much more than the extent of their within-school experience (OECD, 2016; for Germany also see Wild and Esdar, 2014), the extent of individuals’ relevant out-of-school experience could moderate the predictive validity of expectancies and values derived from within-school and out-of-school activities, as well as the stability of initial field-of-study-specific expectancies and values.

Against this backdrop, the goal of the present study is threefold. The first is to examine the predictive validity of first-year students’ motivational beliefs derived from their experience in secondary school (i.e., subject-specific motivational beliefs) and their motivational beliefs derived from relevant out-of-school experience in predicting initial field-of-study-specific motivational beliefs. The second is to investigate changes in first-year students’ field-of-study-specific motivation during the first months at university which presumably result from individuals’ experiences with new learning contents. The third goal is to investigate whether the extent of students’ relevant out-of-school experience might moderate the abovementioned processes. In line with previous research (e.g., Guo et al., 2015; Musu-Gillette et al., 2015), this study focuses on academic self-concept (ASC) of ability—individuals’ mental representations of their abilities—as an indicator of expectancy of success (Marsh, 1986, 2007) and on intrinsic task value (ITV)—individuals’ (expected) enjoyment of or interest in a task (Krapp, 1999; Hidi and Harackiewicz, 2000; Ryan and Deci, 2000; see Eccles, 2005, for details on the conceptualization of task value). The sample consists of first-year students studying “business administration,” which includes mathematics, law, economics, and management contents.

### Anticipating Academic Self-concept and Intrinsic Task Value Regarding Novel Academic Tasks

With the expectancy-value model, Eccles (1983, 1994) and Wigfield and Eccles (2000) have developed and validated an integrative theoretical framework to explain students’ educational task choice and academic performance. Its core components, expectancy of success and subjective task value, relate to key theoretical concepts in the literature of motivation in education (Eccles, 2005; Schunk et al., 2014) and directly predict task choice and performance in educational contexts. Beyond that, the model incorporates factors from the individual’s socio-cultural environment and his or her educational biography. The model has been widely used in empirical research to predict students’ course choice in high school and occupational orientations (Eccles, 1994; Bong, 2001; Eccles et al., 2004; Nagengast et al., 2011; Guo et al., 2015), students’ choice of college majors (Musu-Gillette et al., 2015) and students’ career plans during college (Jones et al., 2010).

The present study shifts the focus of attention to the predictors of expectancy of success and task value. According to Wigfield and Eccles (2000), an individual’s expectancies of success and task values are rooted in established self-schemata and affective memories related to his or her personal learning experiences within a content domain (see Wigfield et al., 2006, for a detailed account of the development of expectancies and task values during childhood and adolescence). For example, because students study mathematics over at least 9–10 years of schooling, their mathematics-specific ASC and ITV rely on extensive personal experience with the subject matter. Accordingly, empirical findings demonstrate increasingly stable ASC and ITV across the secondary school years in core subject areas (e.g., Denissen et al., 2007). In addition, students’ experiences from out-of-school activities may contribute to the development of school subject-specific expectancies and values: research from within primary school has presented evidence for a positive effect of the extent of students’ out-of-school science activities on their science-specific expectancies and values (Simpkins et al., 2006).

Most studies of motivation beyond compulsory schooling limit their focus to school-based classifications of subject matters (i.e., “mathematic-intensive and non-mathematic-intensive college majors”; Musu-Gillette et al., 2015; “science” at college; Shernoff and Hoogstra, 2001) or use higher-order classification systems, grouping both school subjects and college majors under broad categories based on Holland’s (1973) typology of interests (Nagy, 2007; Warwas et al., 2009; Volodina and Nagy, 2016), for example. The drawback of these approaches is the loss of information about the specific learning contents. In addition, many study programs, especially more recent and applied ones, cover several groups of subjects if not interests (Schröder, 2015). Hence, such approaches may explain task choice on a high level of abstraction, but they appear rather far from specific educational choices and may not explain why, for example, a student who is interested in mathematics prefers *construction engineering* over *electrical engineering*.

Only recently researchers began investigating task choice motivation via school subject-specific ASC and ITV, and how these predict field-of-study-specific ASC and ITV (Gorges and Göke, 2015; Gorges, 2016a). Reviewing theory and research on the formation of ability beliefs (i.e., expectancy of success reflected by self-concept of ability and self-efficacy; Bandura, 1997; Bong and Skaalvik, 2003; Marsh, 2007) and internal forms of motivation (i.e., intrinsic motivation, interest, and ITV; Ryan and Deci, 2000; Krapp, 2002, 2005; Eccles, 2005), the authors conclude that both constructs are highly subject-specific, and heavily rely on learners’ personal experience with this subject. In other words, expectancy of success and ITV primarily develop from a person’s experience with a subject matter. With respect to higher education, students’ may have gathered experiences that are potentially relevant to their future study program in within-school settings and from out-of-school activities that took place alongside compulsory schooling (e.g., from a side job) or afterward (e.g., during long-term internships or by completing vocational education and training [VET]).

Nevertheless, within-school and out-of-school settings unlikely provide the exact same experience with a learning content than studying at university. In this case, students need to build bridges between what learning contents they already know and what they expect in the new educational context (Gorges and Kandler, 2012). In this regard, perceived similarity between old and new has consistently been brought forward to explain developmental processes. Learned reactions to specific stimuli generalize across similar stimuli (Bierley et al., 1985), research on cognitive processes suggests that new knowledge gradually enters a person’s knowledge base along the lines of similar knowledge already represented (Wittrock, 1974; Anderson, 2010), and research on interest suggests that learners use their interest regarding more abstract topics to evaluate their interest for specific topics (Ainley et al., 2002; Krapp, 2002). Taking the idea of generalization to the field of motivation in educational transitions, Gorges (2016a; Gorges and Göke, 2015) investigated the extent to which school subject-specific self-concepts of ability and ITV differentially predict (i.e., generalize to) field-of-study-specific self-concepts of ability and ITV. As expected, results indicate that the former predicts the latter when the students perceive the learning contents to be similar (e.g., physics-related motivational beliefs predicted motivational beliefs regarding mechanical engineering).

Against this background, the motivational beliefs developed from within-school and out-of-school experiences alike should play a role in anticipating academic self-concepts and task values beyond the high school context. For example, school subjects should be relevant for anticipating field-of-study-specific motivational beliefs only when they are considered to be similar to the field of study (Gorges and Göke, 2015; Gorges, 2016a), and VET in commercial occupations should be relevant for anticipating motivational beliefs regarding business administration, whereas craftsman experience should not. Likewise, with respect to a study program in higher education, academic experience may be more relevant than practical experience. Therefore, the present study extends previous research by considering motivational beliefs developed from relevant out-of-school experience–split into academic and practical experience—as another potentially relevant source of motivational beliefs in addition to students’ motivational beliefs developed from experience with an ostensibly similar school subject (Gorges and Göke, 2015; Gorges, 2016a).

### From Anticipated to Experience-Based Academic Self-concepts and Intrinsic Task Values

Due to accumulation of academic experience across secondary school, older students’ self-concepts and values hardly change. Guo et al. (2015) report highly stable general ASC between early 10th and late 11th grade (*r* = 0.84), and between late 11th grade and 1 year after secondary school graduation (*r* = 0.78). Students’ general school-related ITV was less stable (*r* = 0.63 and *r* = 0.34, respectively). Correlations of subject-specific motivational beliefs between 10th, 11th, and 12th grade in US high school range from *r* = 0.77 to *r* = 0.86 for mathematics-specific ASC, and from *r* = 0.65 to *r* = 0.74 for interest in mathematics (Musu-Gillette et al., 2015). Covering a period of 2 years, longitudinal research from upper secondary school in Germany also reported substantially correlated mathematics-specific self-concepts (*r* = 0.63) and ITV (*r* = 0.58; Köller et al., 2006). Obviously, ASC appear particularly stable when measured as subject-specific and across shorter periods of time, whereas ITV is a little less stable, but also shows high stability when measured as subject-specific and across shorter periods of time.

Given that both ASC and ITV primarily rely on an individual’s personal experience with a particular learning content (Wigfield and Eccles, 2000; Gorges and Göke, 2015; Gorges, 2016a), individuals’ should review and—as necessary—revise their motivational beliefs once personal experiences with novel learning contents take place. Consequently, while processing their new academic experiences. Hence, students potentially adjust their initially anticipated motivational beliefs. Building on their anticipated ASC and ITV and their personal learning experience, students may confirm their initial motivational beliefs (i.e., the newly made personal experience matches what they expected based on their initial motivational beliefs), or increase or decrease their expectancies and task values (i.e., the newly made personal experiences are better or worse than they expected). In line with these assumptions, findings reported by Guo et al. (2015) reveal decreases in ASC and ITV stability (*r* = 0.78 to *r* = 0.34, respectively) when participants had left secondary school. Similarly, in a study on students training to be teachers, Rösler et al. (2013) report relatively small stability coefficients for students’ pedagogical knowledge-specific ITV over the first year at university (*r* = 0.35), and Jones et al. (2010) observed significant decreases of engineering-specific expectancy and value over the first semester at university.

Despite the importance attached to motivational beliefs for first-year students’ adaptation, performance, and retention in higher education (e.g., Bong, 2001; Chemers et al., 2001; McKenzie and Gow, 2004; Mattern and Shaw, 2010; Vuong et al., 2010; Krumrei-Mancuso et al., 2013), empirical findings regarding changes in first-year students’ initial field-of-study-specific motivational beliefs are rare (but see Jones et al., 2010; Rösler et al., 2013). Therefore, the present study addresses changes in two key motivational constructs, field-of-study-specific ASC and ITV, across students’ first months in higher education. In addition, because motivational beliefs relying on within-school and out-of-school experience are assumed to primarily predict anticipated field-of-study-specific motivational beliefs, it investigates the role of anticipated field-of-study-specific motivational beliefs as a mediator in predicting experience-based field-of-study-specific motivational beliefs.

### The Moderating Role of Extensive Relevant Out-of-School Experience

Considering students’ relevant out-of-school experience as a source of information for their field-of-study-specific motivational beliefs points to systematic differences with respect to the extent of students’ within-school versus out-of-school experiences. Because most developed countries have a fixed compulsory schooling period and specific entry requirement for higher education, the extent of students’ within-school experience should be rather similar, whereas students’ out-of-school experience might vary substantially, especially in the light of the expansion of lifelong learning (OECD, 2016). Hence, assuming that self-concepts of ability and task values derived from out-of-school experiences contribute to students’ anticipation of their field-of-study-specific self-concepts of ability and task values leads to the assumption that the extent of students’ out-of-school experience may act as a moderator. In other words, students arriving at university with relevant out-of-school experience (when the study program is not part of the secondary school curriculum in particular) may benefit from extensive out-of-school experience because they have more personal experiences to rely on when anticipating field-of-study-specific motivational beliefs. By contrast, others may only have a vague idea of what will be the learning content in their study program (Moogan et al., 1999; Schröder, 2015), which may increase reliance on school subject-specific experiences when anticipating motivation. For example, students who have gathered experiences that are potentially relevant to their future study program during internships or VET where they learn what more occupational-relevant tasks are like may expect these tasks in occupation-relevant university tracks. Hence, they may consider ASC and ITV formed in such out-of-school settings to be more important than students without such experiences. The extent of students’ relevant prior experience may also affect changes in self-concepts of ability and task values because students with extensive experience from relevant out-of-school activities may better—or more accurately—anticipate their study-specific ASC and ITV.

In support of these assumptions, research on non-traditional students—i.e., students that are older than the average school-leaver and, therefore, have presumably more out-of-school experience (Donaldson and Townsend, 2007; Langrehr et al., 2015)—does in fact document significant differences in non-traditional students’ versus traditional students’ motivation (Landrum et al., 2000; McKenzie and Gow, 2004; Bye et al., 2007; Tilley, 2014). However, because this line of research typically identifies non-traditional students based on student age, it is unclear to what extent these students dispose of *relevant* out-of-school experience, and whether motivational beliefs rooted in their out-of-school experience actually play a role in their field-of-study-specific motivation. To illustrate this gap in the literature, a non-traditional student may be highly motivated because he or she has previously been working in his or her area of study (e.g., a commercial clerk now studies business administration), or because he or she has finally decided to leave his or her previous occupation behind to pursue his or her core interests (e.g., a carpenter decides to study education science; Gorges, 2016b).

Using German samples, Wild and Zimmermann (2005) and Jürgens (2014) found that students with (versus without) relevant out-of-school experience from VET have a higher interest in their study matter. Academic self-concepts and task values developed from these experiences may contribute to students’ anticipation of ASC and ITV regarding their field of study, for example, by complementing school-based ASC and ITV. However, general motivation differences between traditional and non-traditional students may be explained by the non-traditional students being older and more mature, therefore knowing better where they want to go in life. In fact, none of these studies have directly linked ASC and ITV established through out-of-school activities to field-of-study-specific motivational beliefs.

Overall, research on mature students has suggested that relevant out-of-school experience gathered between graduation from high school and entering university may promote students’ field-of-study-specific motivation. The present study seeks to scrutinize the role of the extent of students’ relevant out-of-school experience as a moderator for predicting field-of-study-specific self-concept of ability and task value from self-concept of ability and task value related to within-school and out-of-school experience, and changes in field-of-study-specific self-concept of ability and task value across the first months in higher education.

### The Present Study

The present study tested hypotheses derived from expectancy-value theory regarding first-year students’ initial field-of-study-specific ASC and ITV and change in ASC and ITV over the first few months in higher education when the study program does not correspond to a school subject. In addition, it addressed the potentially moderating role of the extent of students’ relevant out-of-school experience. Data stems from a third-party funded research project described previously (Gorges and Göke, 2015; Gorges, 2016a). Extending existing analyses, the present study used a second measurement point and a different set of variables. The field of study in this research was business administration.

The study aimed at three goals. The first goal concerned first-year students’ anticipation of field-of-study-specific motivational beliefs. Given the subject-specific structure of both ASC and ITV, established ASC and ITV related to learning contents perceived to be similar to or relevant for business administration were hypothesized to predict anticipated business administration ASC and ITV. In other words, ASC and ITV regarding relevant within-school and out-of-school experience were expected to contribute to students’ initial anticipation of field-of-study-specific ASC and ITV. Based on previous work, mathematics was chosen as a relevant school subject (Gorges and Göke, 2015; Gorges, 2016a). Out-of-school experience referred to students’ experience from previous studies in higher education, from long-term internships, from company-based vocational training, or from attending vocational school. It was further differentiated into academic and practical experience. In addition, students’ initial business administration-specific ASC/ITV was expected to fully mediate effects of their pre-study ASC/ITV related to mathematics, and practical and academic out-of-school experience on later business administration-specific ASC/ITV.

Hypothesis 1: Students’ ASCs/ITVs regarding mathematics, academic and practical out-of-school experience predict students’ anticipated ASC/ITV regarding business administration at the beginning of their study program.

The second goal was to shed light on changes in students’ business administration-specific ASC and ITV during their initial study phase in higher education. Given that students’ initial ASC/ITV is merely anticipated, both were expected to change subject to students’ personal experience with business administration. Hence, stability coefficients for school subject-specific ASC and ITV have been found to be quite high over short periods of time in within-school longitudinal studies (*r* = 0.86 for mathematics-specific ASC and *r* = 0.74 for mathematics-specific ITV; Musu-Gillette et al., 2015), whereas stability coefficients of ASC/ITV at the beginning of a study program should be markedly lower.

Hypothesis 2: Stability coefficients of students’ initial ASC/ITV are lower than stability coefficients in mathematics-specific ASC/ITV at the end of secondary school as reported by Musu-Gillette et al. (2015).

The third goal was to test whether the extent of students’ relevant out-of-school experience in terms of a dichotomous distinction (i.e., extensive versus little to none) would moderate the predictive validity of mathematics-specific and out-of-school experience-specific ASC/ITV regarding anticipated business administration-specific ASC/ITV and its stability over the initial study phase. ASC/ITV related to out-of-school experience were expected to be stronger predictors for students with (versus without) extensive relevant out-of-school experience indicated by study experience, VET and/or long-term internships. Further, these students were expected to have a more accurate anticipation of their field-of-study-specific ASC/ITV, that is, their ASC/ITV should be more stable over time.

Hypothesis 3: The hypothesized relations between ASC/ITV related to relevant mathematics, out-of-school experience and business administration differ as a function of the extent of students’ relevant out-of-school experience.

In sum, the present study extends the literature on expectancy-value theory in three ways. It explicitly addresses ASC and ITV developed from out-of-school-experience as antecedents of first-year students’ field-of-study-specific ASC and ITV when facing a novel academic task, it adds to the scarce empirical findings on motivational change during the initial study phase, and it compares groups of students that have little versus extensive out-of-school experience considered relevant for the respective field of study (here: business administration).

## Materials and Methods

### Procedure

Participants were recruited from universities of applied sciences in Germany that typically have student bodies including a substantial number of those with extensive relevant out-of-school experience relevant for their field of study from company-based VET, school-based VET and/or extended internships (partly because some of these institutions require practical experience as an admission criterion; Willich et al., 2011). Recruitment of participants was limited to study programs labeled *business administration* (in German “Betriebswirtschaft” or “Betriebswirtschaftslehre”). Similar study programs with different labels, such as *management* or *economics* were excluded. Eight universities of applied sciences, selected based on having a study program labeled *business administration* with as many first-year students as possible, were contacted and six consented to cooperate.

As the target participants had not yet started their study programs, online data collection was used to provide low-threshold access to participation in the study. This procedure, however, is typically susceptible to low response rates. Hence, to maximize the initial sample of incoming first-year business administration students for the fall semester, the universities’ executives were contacted the preceding spring and asked to support the project’s data collection. The universities sent out invitations to their newly enrolled first-year students to participate in the study twice, by email or by post. In addition, the researchers visited five universities to announce the project in person during the first weeks of the semester.

Students were surveyed at the beginning of their study program and again 3–4 months afterward (i.e., toward the end of their first semester). The online survey was administered using EFS survey software. In the first survey (t1, September to beginning of October 2013), participants gave their email addresses (kept separate from the data files) to be contacted for the follow-up survey (t2, mid-December 2013 to mid-February 2014). Participants’ data were linked across measurement points using a personal code, which could be reproduced by the participants, but did not disclose their identities.

The study was carried out in accordance with the recommendations of the German Psychological Association’s guidelines for conducting research with human subjects. All human subjects were adult and consented in participation in the study and processing of their data prior to participation. No subjects were harmed or deceived during the study.

### Participants

Approximately 1,200 students received the invitation to participate in the study and 408 started the survey; hence, the response rate was about 34%. The sample composition matches those in other empirical studies of student bodies from this field of study at universities of applied sciences in terms of gender and prior experience (Willich et al., 2011). Participants with missing data on all variables were excluded from the analyses. Due to technical problems, codes and sociodemographic information from 17 persons at t2 were not recorded and their t2 data were excluded leaving *N* = 341 participants in the final sample. In all, 41% of these participants did not take part in the second survey. Moreover, seven participants did not report age and gender. Attrition was within the normal range compared to similar studies (e.g., Rösler et al., 2013). **Table 1** shows descriptive statistics for the samples at t1 and t2.

**Table 1:** Participant characteristics.

	t1 (*n* = 341)	t2 (*n* = 200)
Age	*M* = 21.6	*M* = 21.4
	*SD* = 3.56	*SD* = 3.53
	Range 17–43	Range 17–43
Gender	57.5% female	62.9% female
Prior experience	190 (55.7%)	115 (57.4%)
of which:		
Prior university study	11	6
Company-based VET	124 (97% completed)	78
School-based VET	39 (95% completed)	23
Internship (>6 weeks)	52	28

*VET, vocational education and training*.

To investigate the hypothesized moderating effect of the extent of relevant out-of-school experience, participants who responded to the questions regarding out-of-school experience were split into two groups, the *with-experience group* (*n*_with_ = 190) and the *without-experience group* (*n*_without_ = 151). Participants were assigned to the with-experience group if they reported at least one of the following: relevant study experience, company-based VET, school-based VET, long-term internships, which in Germany typically refers to internships of more than 6 weeks. The two groups differed in mean age (with: *M* = 19.98, *SD* = 2.37; without: *M* = 23.54, *SD* = 3.86) because relevant out-of-school experience typically leads to students entering higher education later in life. The share of female students was larger in the without-experience group (62.9% versus 51.4%).

### Measures

#### Structure of the Online Survey

The survey first covered students’ socio-demographic characteristics, including their relevant out-of-school experience and measures to assess ASC and ITV regarding relevant out-of-school experience, which were presented on the same survey page (for details see section “Tapping Motivation Regarding Out-of-School Experience”). Next, the following instructions appeared on top of the page containing ASC/ITV measures regarding school subjects: “We will now ask about your ability beliefs and values attached to a range of fields of study and school subjects. Please read carefully through the following statements. Indicate how true the statement is for yourself separately for each field of study or school subject. Each statement contains “…”. Please insert the field of study or school subject given as a title of the respective statements in your mind when you respond.” In addition, participants were actively encouraged to anticipate their responses although they may have had no personal experience with the learning content. The instructions emphasized that the study focused on the participants’ personal appraisals and that there were not correct or incorrect answers. Below these instruction, ASC and ITV measures regarding mathematics, German, history, and physics were presented in terms of a matrix in varying order. The general structure of these pages comprised four areas to present the items [see sections “Academic Self-concept of Ability (ASC)” and “Intrinsic Task Value (ITV)”]. For example, all statements regarding mathematics could be displayed top left, German top right, history bottom left, and physics bottom right. Measures regarding business administration-specific ASC and ITV were presented in an analogous way alongside items referring to engineering, linguistics, and sociology.

#### Tapping Motivation Regarding Out-of-School Experience

At the beginning of the survey, participants indicated whether they had relevant out-of-school experience from, for example, long-term internships or VET. The instructions then introduced the distinction between practical and academic experience deemed relevant for business administration: “Depending on the kind of previous experience, practical (e.g., internships) and academic (e.g., vocational school) experience may be distinguished. In the following, we would like to learn more about your out-of-school experiences relevant for your study program and your beliefs about your practical and academic knowledge and competences. Please refer to tasks and activities related to business administration when answering the following questions.” Participants were then asked to rate the same items used to assess mathematical and business administration-specific ASC and ITV [see sections “Academic Self-concept of Ability (ASC)” and “Intrinsic Task Value (ITV)”] with respect to their practical and academic business administration-related experience.

#### Academic Self-concept of Ability (ASC)

Academic self-concept was measured using four items from Dickhäuser et al. (2002), adapted in this survey to reference mathematics, relevant practical or academic out-of-school experience, and business administration, respectively. The four items read: ‘I consider my aptitude for “[e.g., mathematics]” to be high,’ ‘I can deal with the requirements in “[e.g., mathematics]” better than others,’ ‘My ability regarding “[e.g., mathematics]” is high,’ and ‘I am good at “[e.g., mathematics]”.’ Participants responded to all items on a four-point Likert-type scale (1 = *absolutely not true*, 2 = *rather not true*, 3 = *somewhat true*, and 4 = *absolutely true*). Means and standard deviations can be obtained from **Table 2**. Internal consistency was high across all ASC scales (see **Table 4**).

**Table 2:** Means (M) and standard deviation (SD) of each variable for the full sample, for the without-experience group, and for the with-experience group.

Variable	Full sample *M (SD)*	With-experience group *M (SD)*	Without-experience group *M (SD)*
ASC mathematics	2.80 (0.77)	2.70 (0.76)	2.93 (0.77)
ITV mathematics	2.84 (0.76)	2.79 (0.76)	2.89 (0.75)
ASC practical experience	3.08 (0.50)	3.21 (0.45)	2.90 (0.51)
ITV practical experience	3.32 (0.54)	3.42 (0.47)	3.19 (0.60)
ASC academic experience	2.92 (0.47)	2.90 (0.46)	2.94 (0.49)
ITV academic experience	2.98 (0.53)	3.03 (0.51)	2.92 (0.56)
ASC business administration t1	3.13 (0.52)	3.21 (0.49)	3.03 (0.54)
ITV business administration t1	3.39 (0.53)	3.45 (0.50)	3.31 (0.55)
ASC business administration t2	3.01 (0.56)	3.09 (0.52)	2.90 (0.59)
ITV business administration t2	3.22 (0.53)	3.26 (0.51)	3.17 (0.56)

*ASC, academic self-concept; ITV, intrinsic task value; participants have been assigned to the with-experience group based on any one of the following: experience from studying, company-based or school-based vocational training, or long-term internship (>6 weeks) in the field of business administration, commerce, or economics*.

#### Intrinsic Task Value (ITV)

Intrinsic task value was measured using four items based on Steinmayr and Spinath (2010), also adapted to reference mathematics, relevant practical or academic out-of-school experience, or business administration, respectively. The four items read: ‘I have fun doing “[e.g., mathematics]”,’ ‘I think “[e.g., mathematics]” is interesting,’ ‘I like being engaged in “[e.g., mathematics]”,’ and ‘I enjoy dealing with “[e.g., mathematics]”.’ Participants responded to all items on a four-point Likert-type scale (1 = *absolutely not true*, 2 = *rather not true*, 3 = *somewhat true*, and 4 = *absolutely true*). Means and standard deviations can be obtained from **Table 2**. Internal consistency was high across all ITV scales (see **Table 4**).

### Statistical Analyses

#### Preliminary Analyses

Preliminary analyses covered survey attrition using Pearson’s χ^2^-test and multivariate analyses of variance (MANOVA).

#### Confirmatory Factor Analyses

**FIGURE 1:**
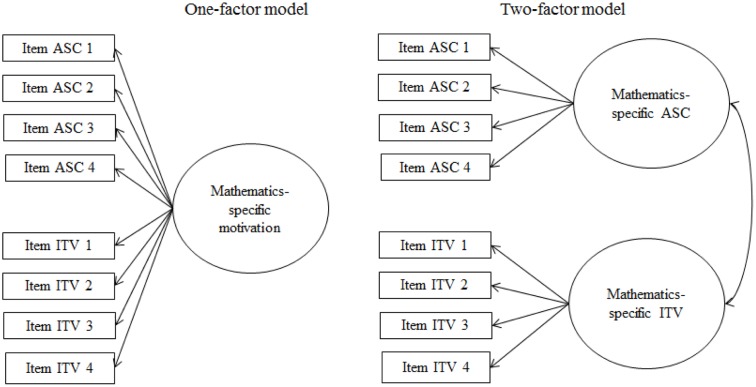
Models of confirmatory factor analyses to test measurement models and discriminant validity of mathematics-specific ASC and ITV. ASC, academic self-concept; ITV, intrinsic task value.

**FIGURE 2:**
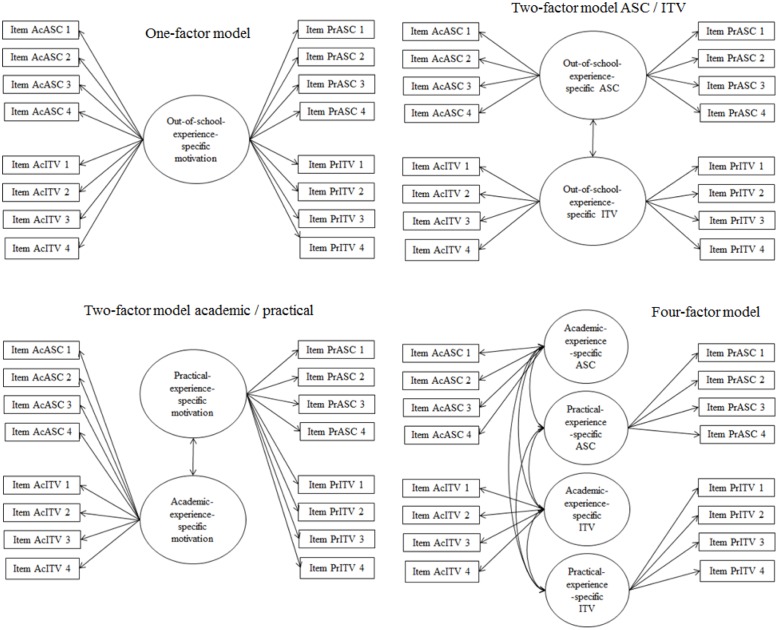
Models of confirmatory factor analyses to test measurement models and discriminant validity of experience-specific ASC and ITV. ASC, academic self-concept; ITV, intrinsic task value.

**FIGURE 3:**
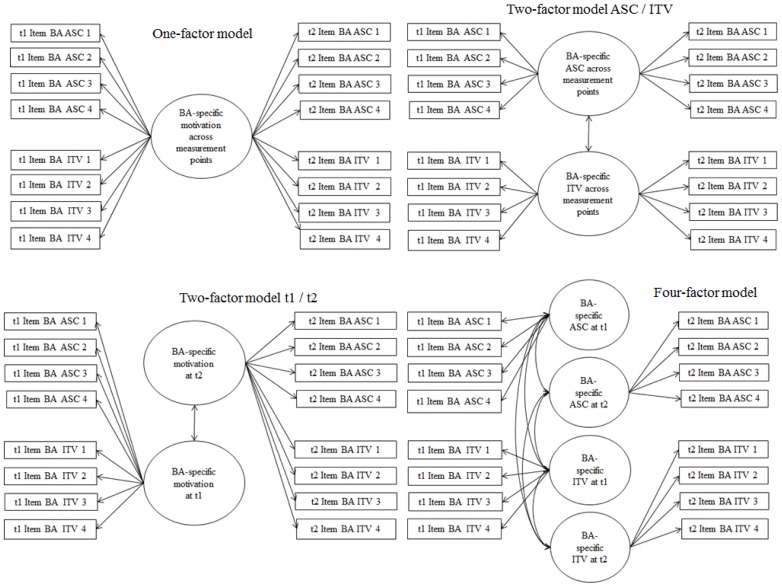
Models of confirmatory factor analyses to test measurement models and discriminant validity of business administration-specific ASC and ITV. ASC, academic self-concept; ITV, intrinsic task value; BA, business administration.

Multiple confirmatory factor analyses tested discriminant validity of the measures and investigated measurement invariance over time. Regarding mathematics, a one-factor model containing all ASC and ITV indicators was compared to the hypothesized two-factor model (see **Figure 1**). Regarding relevant out-of-school experience, a one-factor model containing all indicators for both practical and academic ASC and ITV, a two-factor model distinguishing between indicators related to practical versus academic experience, and a two-factor model distinguishing between ASC and ITV, were compared to the hypothesized four-factor model distinguishing practical ASC, practical ITV, academic ASC, and academic ITV (see **Figure 2**). Similarly, a one-factor model of all business administration-specific ASC and ITV indicators, two two-factor models distinguishing between ASC versus ITV, and t1 versus t2, respectively, were compared to the hypothesized four-factor model (see **Figure 3**). A model with factor loadings of t1 and t2 business administration-specific ASC/ITV items fixed to be equal across measurement points was compared to a model with freely estimated loadings to test the assumption of weak measurement invariance over time. Weak measurement is a necessary prerequisite to draw valid conclusions from structural models across measurement points using equivalent latent variables and items (Meredith, 1993; Steenkamp and Baumgartner, 1998). Bivariate correlations were analyzed based on latent variables.

#### Structural Equation Models

To test Hypothesis 1, directional relations between the constructs were analyzed using structural equation models (SEM) with latent variables. Due to potentially high correlations between ASC and ITV they were analyzed in separate models. Correlations among the predicting variables were allowed. Measures of mathematics-specific, practical and academic experience-specific ASCs and ITV, respectively, were specified to predict t1 business administration-specific ASC (Model 1a) and ITV (Model 2a), respectively. Significant path coefficients were expected for all three predictors (i.e., ASC/ITV regarding mathematics, practical and academic experience). Next, t2 business administration-specific ASC and ITV, respectively, was added to the model (Model 1b/2b) with t1 business administration-specific ASC/ITV mediating the effects of the three predicting variables (i.e., ASC/ITV regarding mathematics, practical and academic experience).

To test Hypothesis 2, effects of the predicting variables mathematics-specific, practical and academic experience-specific ASCs/ITVs on t2 business administration ASC/ITV were fixed to zero. Thus, the path coefficient for t1 business administration-specific ASC/ITV predicting t2 business administration ASC/ITV reflected bivariate relations between these variables. Next, these path coefficients were compared to the respective bivariate correlational coefficients for mathematics-specific ASC/ITV between 11th and 12th grade reported in the literature (mathematics-specific ASC: *r* = 0.857; ITV: *r* = 0.743 in a sample of 421 students; Musu-Gillette et al., 2015) using Fisher’s z-transformation to obtain approximately normally distributed coefficients and 95% confidence intervals. Distinct (i.e., not overlapping) confidence intervals indicate significant differences between coefficients (Eid et al., 2013).

#### Multiple-Group Structural Equation Models

A multiple-group SEM for students with versus without extensive relevant out-of-school experience was specified to investigate potential moderating effects. As preliminary analyses, weak measurement invariance was tested by comparing models with factor loadings constrained to be equal across the two groups (Model 1d/2d) against models where all factor loadings were freely estimated (Model 1c/2c). Weak measurement is a necessary prerequisite to draw valid conclusions from comparisons of structural models across groups (Meredith, 1993; Steenkamp and Baumgartner, 1998). Next, the structural model (i.e., all regression paths) was constrained to be equal across groups (Model 1e/2e) and model fit was compared to the less constrained model to assess the moderation hypothesis. In each case, significant changes in model fit to the worse would indicate non-invariance across groups regarding the measurement model and the structural model, respectively.

#### Evaluation of Model Fit

All models were fitted to the data using R (R Core Team, 2015) and the lavaan package (Rosseel, 2012) using robust maximum likelihood estimation. Model fit was evaluated based on the comparative fit index (CFI; acceptable fit > 0.90, good fit > 0.95), the root mean square error of approximation (RMSEA; acceptable fit < 0.08, good fit < 0.05), and the standardized root mean square residual (SRMR; acceptable fit < 0.10, good fit < 0.05; Schermelleh-Engel et al., 2003). Changes in model fit were evaluated based on the criteria proposed by Cheung and Rensvold (2002) and Chen (2007) with a decrease in CFI > 0.01 and an increase in RMSEA > 0.015 indicating significant changes in model fit.

## Results

### Preliminary Analysis

Comparing the subsample of retained participants to those who dropped out at t2 demonstrates that the proportion of females was significantly higher at t2 [49.6% versus 62.9%, χ^2^(1) = 5.86, *p* < 0.05]. However, MANOVA indicated no main effect of dropout on the t1 variables [*F*(8,303) = 0.521, *p* = 0.840]. Thus, attrition did not appear to bias the findings.

### Measurement Models and Bivariate Correlations of Latent Variables

Results for all measurement models are summarized in **Table 3**. All factor loadings were high (λ > 0.60) and significant (*p* < 0.05). As expected, models containing theoretically expected factors—i.e., two factors for mathematics-specific ASC and ITV, four factors for business administration ASC and ITV over time, and four factors for practical and academic experience-specific ITV and ASC—showed superior fit over models that collapsed these constructs into fewer factors. Thus, results demonstrate that all measurement instruments discriminate well between the respective constructs and show at least good fit to the data. In addition, factor loadings for ASC and ITV regarding business administration were invariant across time. Hence, all measurement models may be used as expected in subsequent analyses.

**Table 3:** Tests of measurement models.

	χ^2^	*df*	*p*	CFI	ΔCFI^a,b^	RMSEA	ΔRMSEA^b^	SRMR
**ASC and ITV mathematics**								
One-factor model	125.623	9		0.935	*-0.59*	0.199	*+0.133*	0.036
Two-factor model^a^	19.464	8	0.013	0.994		0.066		0.013
**ASC and ITV prior experience**								
One-factor model	875.669	54	<0.001	0.480	*-0.485*	0.215	*+0.156*	0.190
Two-factor model ASC/ITV	818.897	53	<0.001	0.515	*-0.450*	0.210	*+0.151*	0.191
Two-factor model academic/practical	203.247	53	<0.001	0.905	*-0.060*	0.093	*+0.034*	0.060
Four-factor model^a^	102.759	48	<0.001	0.965		0.059		0.045
**ASC and ITV business administration**								
One-factor model	596.422	54	<0.001	0.728	*-0.237*	0.172	*+0.107*	0.111
Two-factor model ASC/ITV	508.601	53	<0.001	0.771	*-0.193*	0.159	*+0.094*	0.119
Two-factor model t1/t2	301.169	53	<0.001	0.876	*-0.088*	0.118	*+0.053*	0.065
Four-factor model^a^	117.584	48	<0.001	0.964		0.065		0.043
Four-factor model loadings invariant over time	122.011	52	<0.001	0.965	+0.001	0.063	-0.002	0.050

*ASC, academic self-concept of ability; ITV, intrinsic task value; ^a^Hypothesized measurement model; ^b^Changes in model fit in comparison to the respective hypothesized model; significant changes in model fit (Cheung and Rensvold, 2002; Chen, 2007) are printed in italics*.

Bivariate correlations of latent variables showed a number of significant relations (see **Table 4**). As expected, ASC and ITV regarding specific learning contents or areas of experience, respectively, were highly correlated and predictors from one domain relate to the outcome measures of this study in a similar way. These results emphasize potential multicollinearity problems that could arise when specifying a model that includes all variables. In support of theoretical assumptions, all but one predictor (mathematics-specific ITV and t2 business administration-specific ITV) correlate significantly to the dependent variables.

**Table 4:** Internal consistency of manifest scales and bivariate correlations of latent scale scores.

	α	(2)	(3)	(4)	(5)	(6)	(7)	(8)	(9)	(10)
ASC mathematics (1)	0.93	**0.865**	0.075	**0.152**	**0.433**	**0.245**	**0.336**	**0.294**	**0.240**	**0.161**
ITV mathematics (2)	0.94		0.024	**0.218**	**0.327**	**0.357**	**0.352**	**0.376**	**0.206**	0.143
ASC practical experience (3)	0.77		–	**0.759**	**0.171**	0.102	**0.453**	**0.317**	**0.369**	**0.210**
ITV practical experience (4)	0.86			–	**0.145**	**0.150**	**0.350**	**0.447**	**0.391**	**0.321**
ASC academic experience (5)	0.76				–	**0.804**	**0.593**	**0.465**	**0.338**	**0.252**
ITV academic experience (6)	0.86					–	**0.465**	**0.493**	**0.230**	**0.243**
ASC business administration t1 (7)	0.85						–	**0.763**	**0.560**	**0.438**
ITV business administration t1 (8)	0.90							–	**0.573**	**0.610**
ASC business administration t2 (9)	0.87								–	**0.852**
ITV business administration t2 (10)	0.87									–

*ASC, academic self-concept; ITV, intrinsic task value; significant coefficients are printed in bold (*p* < 0.05)*.

### Structural Equation Models (SEM)

Model fit of all SEM was at least acceptable (see **Table 5**). Correlations between predictors were mostly significant and ranged between *r* = 0.07 (*ns*) and *r* = 0.43 (*p* < 0.05). In Model 1a, practical experience-specific ASC (β = 0.37, *p* < 0.05) and academic experience-specific ASC (β = 0.49, *p* < 0.05) but not mathematics-specific ASC (β = 0.10, *p* = 0.11) significantly predicted students’ initial (t1) business administration-specific ASC. Hence, mathematics-specific ASC did not predict business administration-specific ASC when ASC regarding relevant out-of-school experience is considered simultaneously. Turning to the analogous model for ITV (Model 2a), practical experience-specific ITV (β = 0.36, *p* < 0.05), academic experience-specific ITV (β = 0.38, *p* < 0.05), and mathematics-specific ITV (β = 0.16, *p* < 0.05) significantly predicted t1 business administration-specific ITV. With the exception of the non-significant path from mathematics-specific ASC these results support Hypothesis 1.

**Table 5:** Model fit.

	χ^2^	*df*	*p*	CFI	RMSEA [90% CI]	SRMR
**ASC models**						
Model 1a	214.677	98	<0.001	0.946	0.059 [0.049–0.069]	0.048
Model 1b	319.601	160	<0.001	0.939	0.054 [0.046–0.062]	0.055
Model 1c	498.200	320	<0.001	0.934	0.057 [0.048–0.066]	0.066
Model 1d	512.477	335	<0.001	0.934	0.056 [0.046–0.054]	0.069
Model 1e	516.621	342	<0.001	0.935	0.055 [0.045–0.064]	0.072
**ITV models**						
Model 2a	122.479	98	<0.001	0.991	0.027 [0.007–0.040]	0.031
Model 2b	174.465	160	<0.001	0.995	0.016 [0.000–0.030]	0.033
Model 2c	399.165	320	<0.001	0.976	0.038 [0.025–0.049]	0.052
Model 2d	413.552	335	0.001	0.976	0.037 [0.024–0.048]	0.054
Model 2e	426.514	342	0.001	0.974	0.038 [0.025–0.049]	0.068

**FIGURE 4:**
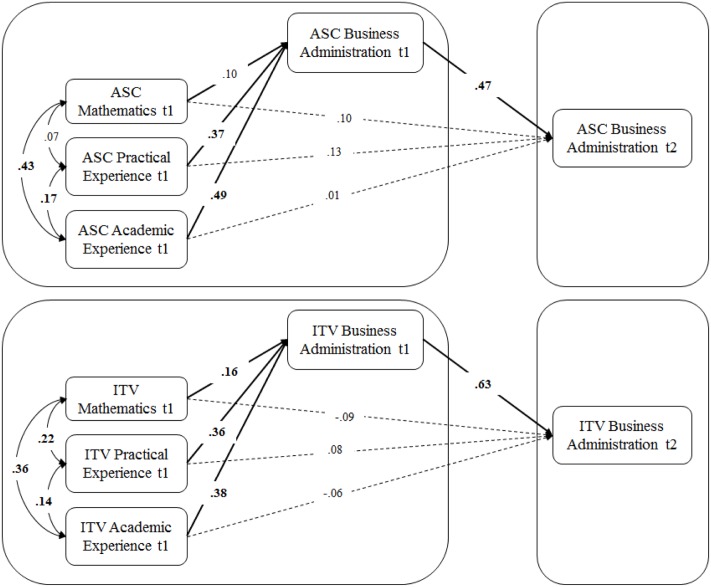
Structural equation models (SEM) of predictors of first-year students’ academic self-concept and intrinsic task value, respectively, and stability over the first semester. ASC, academic self-concept; ITV, intrinsic task value; significant coefficients (*p* < 0.05) are printed in bold, for further description see text. AcASC, academic experience-specific ASC; PrASC, practical experience-specific ASC; AcITV, academic experience-specific ITV; PrITV, practical experience-specific ITV.

*ASC, academic self-concept of ability; ITV, intrinsic task value; Model 1a: Students’ academic self-concepts of ability related to mathematics, practical and academic field-of-study-specific experience predicting students’ initial business administration-specific academic self-concept; Model 1b: Model 1a including business administration-specific academic self-concept after the initial study phase with initial business administration-specific academic self-concept mediating the effects (see **Figure 4**, top model); Model 1c: Model 1b specified as a multiple-group model for students without/with substantial prior experience; Model 1d: Model 1c with factor loadings constrained to be equal across groups (weak measurement invariance); Model 1e: Model 1d with structural weights constrained to be equal across groups; Model 2a: Students’ intrinsic task value related to mathematics, practical and academic field-of-study-specific experience predicting students’ initial business administration-specific intrinsic task value; Model 2b: Model 2a including business administration-specific intrinsic task value after the initial study phase with initial business administration-specific intrinsic task value mediating the effects (see **Figure 4**, bottom model); Model 2c: Model 2b specified as a multiple-group model for students without/with substantial prior experience; Model 2d: Model 2d with factor loadings constrained to be equal across groups (weak measurement invariance); Model 2e: Model 2d with structural weights constrained to be equal across groups*.

Adding t2 business administration-specific ASC (Model 1b) and t2 business administration-specific ITV (Model 2b), respectively, resulted in equally well-fitting models. Both mediation models are depicted in **Figure 4**. As expected, t1 business administration-specific ASC fully mediated effects of both practical and academic experience-specific ASC. Similarly, effects of all three predicting variables on t2 business administration-specific ITV were fully mediated by t1 business administration ITV.

To test Hypothesis 2, direct effects between mathematics-specific, practical and academic experience-specific ASC/ITV on t2 business administration-specific ASC/ITV were fixed to zero, leaving the path between t1 and t2 business administration-specific ASC/ITV to indicate the stability of business administration-specific ASC/ITV in terms of a bivariate relation. Because all paths fixed to zero had been non-significant, model fit did not change significantly compared to the full models 1b and 2b [ASC: χ^2^(*df*) = 322.252(163); Δχ^2^(*df*) = 2.65(3); CFI = 0.940; ΔCFI = +0.001; RMSEA = 0.054 [CI = 0.045–0.062]; ΔRMSEA = 0.000; SRMR = 0.058; ITV: χ^2^(*df*) = 176.935(163); Δχ^2^(*df*) = 2.47(3); CFI = 0.996; ΔCFI = +0.001; RMSEA = 0.016 [CI = 0.000–0.030]; ΔRMSEA = 0.000; SRMR = 0.041].

Next, Fisher’s *z*-values and its 95% confidence intervals were calculated for the stability coefficients from this study and from the Musu-Gillette et al. (2015) study to examine potential overlap of the respective confidence intervals, which would indicate non-significant differences. The stability coefficient for business administration-specific ASC (β = 0.575; *z* = 0.655; CI = 0.515–0.795) was significantly lower than the stability coefficient reported by Musu-Gillette et al. (2015) between 11th and 12th grade in secondary school (β = 0.857; *z* = 1.282; CI = 1.186–1.378). Similarly, the stability coefficient for ITV (β = 0.595; *z* = 0.685; CI = 0.545–0.825) was significantly lower [Musu-Gillette et al. (2015) ITV: β = 0.743; *z* = 0.957; CI = 0.861–1.053] as well. Thus, these results support Hypothesis 2.

### Multiple-Group Structural Equation Models (MG-SEM)

The hypothesized moderating effect of the extent of students’ relevant out-of-school experience was investigated by multiple-group SEM. Model fit can be obtained from **Table 5** (Model 1c-e/2c-e). The first analytic step was fixing all factor loadings to be equal across groups and inspecting potentially significant changes in model fit, which would indicate that the assumption of weak measurement invariance does not hold. As can be seen in **Table 5**, model fit did not deteriorate significantly for the ASC model [Δχ^2^(*df*) = 14.28(15), ΔCFI = 0.000, ΔRMSEA = -0.001] or the ITV model [Δχ^2^(*df*) = 14.39(15), ΔCFI = 0.000, ΔRMSEA = -0.001]. Hence, valid results can be obtained from the subsequent model comparisons across groups.

**FIGURE 5:**
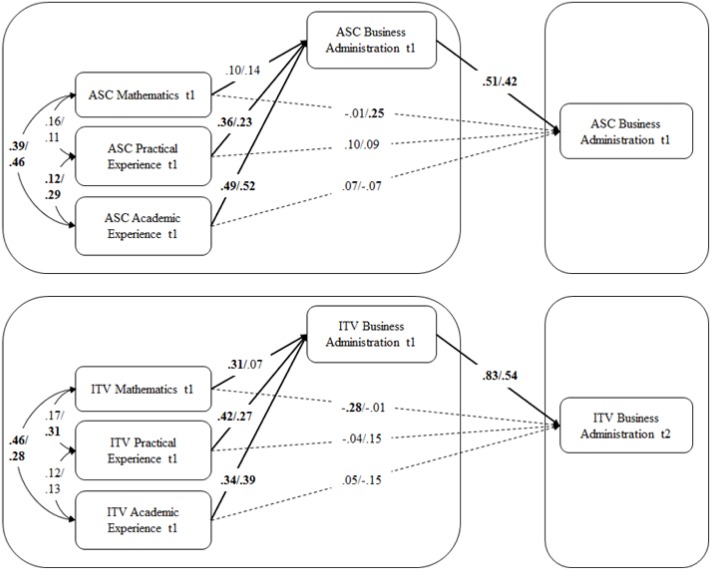
Multiple-group SEM of predictors of first-year students’ academic self-concept and intrinsic task value, respectively, and stability over the first semester for students without/with substantial study-relevant prior experience. ASC, academic self-concept; ITV, intrinsic task value; significant coefficients (*p* < 0.05) are printed in bold, for further description see text.

With respect to path coefficients between ASC constructs in Model 1d (see **Figure 5**), both groups are quite similar. In line with the results reported for the full sample, practical and academic experience-specific ASC significantly predicted t1 business administration-specific ASC, whereas mathematics-specific ASC did not show significant effects. The stability coefficient of business administration-specific ASC is moderate in both groups. Unexpectedly, in the with-experience group, mathematic-specific ASC significantly predicted t2 business administration-specific ASC (β = 0.25, *p* < 0.05) in absence of a direct effect on t1 business administration-specific ASC (β = 0.14, *p* = 0.08). Thus, mathematics-specific ASC only predicted t2 business administration-specific ASC but not t1 business administration-specific ASC.

Results for the ITV constructs revealed a somewhat different pattern. In particular, mathematics-specific ITV significantly predicted t1 business administration-specific ITV only in the without-experience group. In addition, results revealed a significant direct effect of mathematics-specific ITV on t2 business administration-specific ITV, which was negative (β = -0.28; *p* < 0.05). Thus, mathematics-specific ITV contributed positively to t1 business administration ITV but negatively to t2 business administration ITV. Furthermore, students from the without-experience group apparently had a highly stable business administration-specific ITV (β = 0.83; *p* < 0.05).

To scrutinize the statistical significance of these group difference, structural weights were constrained to be equal across groups in both models (Model 1e/2e). Despite the seemingly large differences in effect sizes across groups in the ITV model in particular, model comparison (Model 1/2d versus Model 1/2e) did not justify concluding significant group differences for either the ASC model [Δχ^2^(*df*) = 4.14(7), ΔCFI = 0.001, ΔRMSEA = -0.001] or the ITV model [Δχ^2^(*df*) = 9.96(7), ΔCFI = -0.002, ΔRMSEA = +0.001]. Hence, Hypothesis 3 is not supported by the results in this study.

## Discussion

The present study tested three hypotheses derived from expectancy-value theory regarding the anticipation and initial stability of expectancy of success (i.e., ASC) and ITV for learners facing novel academic tasks. First, business administration first-year students’ ASC and ITV developed from relevant practical and academic out-of-school experience linked to their initial business administration-specific ASC and ITV at the very beginning of the study program, thus tentatively supporting their hypothesized predictive validity. Beyond that, only school-based mathematics ITV was significantly associated with students’ initial business administration-specific intrinsic task, whereas mathematics ASC did not show such an effect. Effects of students’ practical and academic out-of-school experience on their business administration-specific ASC/ITV 3 months after the first measurement were fully mediated by students’ initial business administration-specific ASC/ITV. Second, business administration-specific ASC and ITV show significantly less stability over the first months at university compared to the stability of these constructs over 1 year in secondary school. Third, distinguishing students without versus with extensive prior out-of-school experience (e.g., from vocational training or long-term internships) did not reveal significant moderating effects of the extent of relevant out-of-school experience.

### Anticipation of Field-of-Study-Specific Motivation

Going beyond students’ development throughout primary and secondary school, motivational beliefs have mainly been investigated previously with respect to well-known learning contents, thereby missing the motivational processes that must be in place to evaluate motivational beliefs regarding novel academic tasks with potentially new learning contents when students are adolescents or (young) adults. Building on recent findings regarding the differential predictive validity of school subject-specific motivational beliefs for a range of fields of study (Gorges and Göke, 2015; Gorges, 2016a), the present investigation extends the scope of potential influencing factors to motivational beliefs developed from relevant out-of-school experiences. Results provide evidence that motivational beliefs from out-of-school experiences primarily gathered in between graduation from secondary school and the start of the study program and ostensibly related to a novel set of academic tasks are incorporated in guessing one’s future success and task values—in this case business administration links to prior experiences in VET in commercial occupations. Regarding ASC, taking motivational beliefs related to out-of-school experience into account even supersedes school-based mathematical self-concept’s association with initial business administration-specific self-concept, which was non-significant.

As students grow older and leave high school, take on a job or vocational training, and broaden their experiential horizons, the pool of potentially relevant experiences and related motivational beliefs gets larger and larger. Not surprisingly, individuals will likely use all the experience they have to evaluate their motivation for task engagement. In fact, these practical experiences may be important contributors to future educational expectations. This study thus extends current readings of expectancy-value theory that mostly focused on school-based self-concepts and task values (Shernoff and Hoogstra, 2001; Musu-Gillette et al., 2015), or the sheer number of out-of-school activities (Simpkins et al., 2006). Hence, the antecedents of expectancy and values referred to as “self-concepts” in the model (Wigfield and Eccles, 2000) can be interpreted as established ASC of ability and ITV developed from within-school and out-of-school experiences alike.

### Stability of Field-of-Study-Specific Motivational Beliefs

When students enter higher education and study in a field that is not directly related to their school subjects, they presumably act on anticipated motivational beliefs, which are likely to change through students’ personal experiences with the new learning contents. In line with these assumptions, there seems to be stronger variability in students’ field-of-study-specific ASC and ITV at the beginning of a study program in particular compared to what has been reported from within secondary school studies (Musu-Gillette et al., 2015). The present study thus adds to the scarce empirical evidence on change in motivational beliefs at the beginning of a study program. In line with findings reported by Rösler et al. (2013) and Guo et al. (2015), stability coefficients ranged between *r* = 0.058 for business administration-specific ASC and *r* = 0.60 for business administration-specific ITV. These effect sizes are significantly lower than what has been reported over 1 year in secondary school by Musu-Gillette et al. (2015) and close to what has been reported across 1 year after graduation from secondary school (Guo et al., 2015) or 1 year into a study program for teacher students (Rösler et al., 2013). These results are particularly striking given that—unlike previous studies—the students in our study reported such variability across only 3–4 months. Hence, results support the hypothesis that entering higher education is a potentially volatile transition that leads students’ to change their anticipated field-of-study-specific ASC and ITV based on personal experience with the field of study at the very beginning of their study program (Jones et al., 2010; Rösler et al., 2013).

With respect to the feedback loop incorporated in more recent depictions of the expectancy-value model (e.g., Eccles, 2005), such instable and potentially decreasing motivational beliefs may affect educational task choice, for example, choosing to drop out from college. More specifically, the feedback loop means that each task choice leads to new experience with the chosen task, which, in turn, feed back into the system to channel the next task choice(s). The time period covered in between educational decisions, however, is not limited to school years or years between educational transitions. In higher education, dropout may represent a task choice just as choosing a major is considered an educational task choice. Hence, the motivational processes suggested here—anticipating and revising motivational beliefs—may lead to students’ decisions to stay in or exit higher education.

### The Role of the Extent of Relevant Out-of-School Experience

The overall results from the present study do not warrant the conclusion that the extent of students’ prior practical or academic out-of-school experience significantly affect the relations between predictors of ASC and ITV and field-of-study-specific ASC and ITV at the beginning of the study program, or their stability over the initial study phase. Nevertheless, inspection of the path coefficients for each group yield some interesting results that may be worth speculating about. In particular, the mathematics-specific ASC apparently did not affect t1 business administration-specific ASC but did predict t2 business administration-specific ASC for students with extensive relevant out-of-school experience. This could point to these students revising their initial assumption regarding the relevance of school-based mathematical subject matter after a few months at university. At the beginning, these students may have evaluated their practical and academic experience from out-of-school contexts such as vocational training as being more important than their experience from the secondary school classroom. However, the first weeks of studying business administration may have demonstrated that secondary school mathematics is necessary to pass the courses. Interestingly, the ITV model showed a reversed effect with mathematics-specific ITV predicting initial business administration-specific ITV, whereas mathematics-specific ITV even showed a negative effect on business administration-specific ITV 3–4 months later for students who did not report extensive relevant out-of-school experience. Hence, these students may have relied heavily on the assumption that business administration resembles mathematics classes but then had to acknowledge major differences. Overall, the pattern of results might point to systematic differences in first-semester students’ conception of business administration as a function of the extent of students’ relevant out-of-school experience, which, in turn, may affect cognitive processes to estimate one’s field-of-study-specific ASC and ITV.

### Practical Implications

Based on this study, two aspects of practical significance for task choice in higher and further education emerge that may be particularly relevant for higher education institutions’ efforts to support their prospective and newly arrived students. First, as prospective students draw on a range of prior experiences to anticipate their ASC and ITV for novel learning opportunities, it appears crucial to help them select the experiences that are actually relevant for the novel academic task. For example, information offered by universities could comment on the relations between a study program and typical secondary school subjects made by both experts (i.e., lecturers and professors) and students. Second, although first-year students have more or less voluntarily opted for their study programs and probably begin them quite motivated, their motivational beliefs are fragile and are potentially threatened by early experiences of failure or boredom. Hence, catering for initial dissatisfaction and providing opportunities to gather confidence quickly in one’s academic abilities may be good advice to help students who struggle with the new demands.

### Limitations

The present study has some limitations to interpreting the results. Motivational beliefs considered here were specific to business administration as an overall study program. However, business administration consists of a range of different learning contents. Students probably develop an even more specific ASC and ITV once they get to know these different learning contents. In addition, students also have a general ASC and academic ITV that probably interacts with subject-specific motivational beliefs. The present study considered mathematics-specific self-concept and ITV as school-based predictors for business administration-specific self-concept and ITV. This focus has been derived from previous analyses pointing to mathematics-specific motivational beliefs as relevant predictors for business administration-specific motivation beliefs in particular (Gorges and Göke, 2015; Gorges, 2016a). Nevertheless, the school curriculum likely offers alternative school subjects that students may perceive as similar to business administration and, consequently, use as a reference category (e.g., social sciences). Also, students may have had business administration or economics as a school subject, although this is rare in German general education secondary schools. In addition, the line between students with versus without extensive relevant out-of-school experience may be drawn differently. Finally, a different motivational aspect not found in this study is the utility value related to a study program. For example, even if students’ ITV wavers, they may still focus on occupational goals they want to attain by completing the study program. Such utility value may be linked to both ASC and ITV, and may contribute to their development. In addition, students may reconsider and potentially change their initial utility values throughout their time at university.

Beyond that, several methodological limitations apply. The study had a dropout rate of about 43%. Although analyses suggested that attrition did not bias the findings, testing the hypotheses in a larger sample with less attrition would clearly be desirable. A larger sample would also yield more statistical power for multiple-group analyses, which would increase the probability to detect significant group differences (Marsh et al., 2013). The link between mathematics, practical and academic experience ASC and ITV, and initial business administration ASC and ITV is based on cross-sectional data. Hence, these analyses do not warrant causal conclusions. In addition, the effects size of the stability coefficients in this study were interpreted against results from a longitudinal study from a different country. This was because alternative comparison targets did either report stability across a longer period of time or with respect to more general measures of ASC and ITV. Therefore, a longitudinal study across more measurement points covering students’ late secondary school years and their first years at university would contribute to our understanding of the motivational processes addressed in this study. Finally, although tests of scale reliability and measurement models supports the idea of assessing ASC and ITV with respect to prior practical and academic experience, this is the first study taking this approach.

### Outlook

The present study is one of few studies focusing on changes in students’ motivational beliefs across relatively short periods of time in higher education. In addition, it is—to my knowledge—the first study directly tracing field-of-study-specific motivational beliefs back to motivational beliefs developed from relevant out-of-school activities. The study contributes to the literature by drawing attention to hitherto neglected areas of expectancy-value research, and pinpoints important aspects to be considered when researching educational task choice in higher and further education. The results from this study suggest that, in light of lifelong learning, gaining insight into the anticipation of motivational beliefs seems a promising approach to understanding and fostering learning motivation beyond high school.

Nevertheless, much remains to be seen in how students gather and process information including both the length and contents of previous experiences they draw on when facing novel tasks—academic or not. Beyond self-concept and ITV, future studies considering utility value in particular, in addition to all of the aforementioned, would complement the motivational constructs of focus here. Furthermore, attainment value as well as cost (Eccles, 2005) may also contribute to students’ anticipation and motivation and should be included in future studies.

Motivation in higher education and beyond deserves more theory-driven empirical research to spell out how individuals anticipate and adjust motivation when facing novel tasks, and how they go about searching and using information. Going beyond the expectancy-value model considered here, future research could investigate how individuals undertake searching for information about a learning opportunity both internally—that is, within their memory—and externally (Betsch et al., 2002). Given that choosing a study program in higher education is just one educational task choice in a long sequence of educational task choices (i.e., beginning with choosing a secondary school, majors in high school and so on through further education), research on decision making routines (Betsch et al., 2002) may be useful to contribute to our understanding of educational biographies. Such research will not only contribute to the further development of motivational theory, but also have important implications for practitioners across all kinds of educational institutions.

## Author Contributions

The author confirms being the sole contributor of this work and approved it for publication.

## Conflict of Interest Statement

The author declares that the research was conducted in the absence of any commercial or financial relationships that could be construed as a potential conflict of interest.
